# What drives athletes toward dietary supplement use: objective knowledge or self-perceived competence? Cross-sectional analysis of professional team-sport players from Southeastern Europe during the competitive season

**DOI:** 10.1186/s12970-019-0292-9

**Published:** 2019-06-14

**Authors:** Damir Sekulic, Enver Tahiraj, Dora Maric, Dragana Olujic, Antonino Bianco, Petra Zaletel

**Affiliations:** 1 Faculty of Kinesiology, University of Split, Teslina 6, 21000 Split, Croatia; 2 College Universi Bardhosh, 10000 Prishtina, Kosovo; 3 Department of Psychological, Program in Health Promotion and Cognitive Sciences, Sport and Exercise Research Unit, Pedagogical and Education Sciences, University of Palermo, 90144 Palermo, Italy; 4 Faculty of Food Technology and Biotechnology, University of Zagreb, Zagreb, 10000 Croatia; 5 Faculty of Sport, University of Ljubljana, Ljubljana, 1000 Slovenia

**Keywords:** Dietary supplements, Nutritional supplements, Team sports, Athletes, Knowledge, Effects

## Abstract

**Background:**

Issues related to knowledge of nutrition and dietary supplementation (DS) are understudied in professional athletes. This study aimed to examine the possible association between knowledge of nutrition and DS (KN&DS) and dietary supplement use (DSU) among professional athletes involved in team sports.

**Methods:**

The sample comprised professional team-sport athletes (*N* = 912, age: 22.11 ± 3.37 years, 356 females) involved in four Olympic sports: basketball (*N* = 228), soccer (*N* = 324), volleyball (*N* = 154), and handball (*N* = 206). The participants were tested by previously validated questionnaires to examine their self-perceived competence on nutrition and DS (S/KN&DS), their objectively evaluated (tested) KN&DS (O/KN&DS), sociodemographic and sport-specific variables (predictors), and DSU (criterion). Associations between the predictors and the criterion (No-DSU - Irregular-DSU - Regular-DSU) were determined by multinomial regression analysis for the total sample and separately for the studied sports.

**Results:**

DSU was found to be less prevalent in older and more successful players. The O/KN&DS and S/KN&DS were positively correlated with DSU, but S/KN&DS was a stronger predictor of DSU than O/KN&DS. Sport-specific associations between predictors and criterion were identified, with stronger correlations in sports with a higher prevalence of DSU.

**Conclusions:**

Due to the low correlations between O/KN&DS and S/KN&DS in the studied players, this study highlights the necessity for more frequent monitoring of biomarkers of nutritional status and its usage by coaches and practitioners to provide quantitative instruction.

**Electronic supplementary material:**

The online version of this article (10.1186/s12970-019-0292-9) contains supplementary material, which is available to authorized users.

## Introduction

Nutrition does not compensate for a lack of training or inferior physical abilities, but proper nutritional plans affect both fitness and health in athletes, helping them to make the most of their potential [1–4]. Nutrition and nutritional plans can help athletes withstand consistent intensive training and competition [1, 5]. The high physical demands of training and competition predispose athletes to increasingly rely on nutrition, including the usage of dietary supplements, believing they will get an advantage over the competition, maximize their performance and stay competitive and healthy [6–8]. Dietary supplements (DS) are an overarching term for a wide range of products, including food-based products that involve added nutrients (e.g., sports drinks, protein shakes, fortified foods), essential nutrients in concentrated or isolated form (e.g., essential fatty acids, amino acids vitamins, minerals), botanicals and herbals and specific products with potential for optimization of performance and maintenance of health [7, 9].

In general, the prevalence of dietary supplement use (DSU) in high-level athletes ranges from 40 to 93% [10–14]. In short, 88.4% of Canadian athletes involved in diverse sports have been reported to be DS users. The 5 most frequently used DS reported were vitamin C (6.4%), protein supplements (9.0%), multivitamins and minerals (13.5%), sport bars (14.0%), and sport drinks (22.4%) [10]. A similar prevalence of DSU has been reported for American collegiate/student-level athletes, reporting the most frequent DS usage of vitamins/minerals (73.3%), calorie-replacement drinks (47%), protein supplementation (40.3%), and creatine (31,4%) [15, 16]. Reports on Canadian Olympic athletes showed a somewhat lower prevalence of DSU of 65% [17]. The DSU in European athletes ranges from 70 to 80% in young German and elite Finish athletes [18, 19], 90% in Croatian swimmers [11], > 95% in European tennis players [20] and 55% in rugby players [12].

Factors influencing DSU among athletes have rarely been empirically investigated, especially in elite athletes. Indeed, although proper knowledge on nutrition and DSU, including the information based on high-quality peer reviewed research, should be crucial for safe and effective DSU, this problem is evidently understudied in professional athletes. Specifically, it is generally accepted that athletes consume DSs to improve their recovery and performance and/or to overcome the lack of certain nutrients for specific reasons (i.e., vegetarianism, female athletes during their menstrual cycle) [21–24]. However, due to the competitive spirit of sports, athletes are particularly vulnerable to aggressive DS marketing. Although most dietary supplements are produced and distributed in a proper way, inaccurate labeling of ingredients and lack of evaluation from regulatory agencies are known to be a problem, which sometimes leads to negative health consequences and even positive findings on doping substances [25].

The question that arises is what drives athletes toward DS? In other words, it would be particularly interesting to determine whether DSU in athletes is accompanied by proper knowledge (i.e., knowledge about potential benefits, proper use, and potential side effects). Therefore, the aim of this research was to examine the possible association between knowledge about nutrition and DSs (KN&DS) and DSU among professional athletes involved in team sports. Specifically, KN&DS was observed from two perspectives: (i) objectively evaluated the level of knowledge about nutrition and DS and (ii) self-perceived competence about nutrition and DS. The main hypothesis of the study is that KN&DS is positively correlated with DSU in professional team-sport athletes.

## Materials and methods

### Design and participants

The participants in this cross-sectional study were professional team-sport athletes (*n* = 912, age: 22.11 ± 3.37 years, 356 females) involved in four Olympic sports: basketball (*n* = 228), soccer (*n* = 324), volleyball (*n* = 154), and handball (*n* = 206). All players were members of teams participating at the highest competitive level in Croatia and Kosovo during the competitive season of 2016/2017, and all participants were 18+ years of age at the time of testing. Teams were selected randomly, and players were asked to participate in the study by the national sports federations. For the purpose of this study, it is important to note that teams/athletes observed in this study were not supported and/or sponsored by companies related to DS manufacturing and/or distributing.

## Variables and testing

Although there are various validated questionnaires aimed at evaluating the topics we examined in this study, we used measurement tools that were previously used and validated in evaluating the problem of DS and related factors in athletes from Southeastern Europe [11, 26].

All participants were tested with questionnaires examining (i) gender, (ii) age in years, (iii) highest achieved competitive result in their sport (four-point scale including “participation at a national-level competition”, “participation in national-level finals (play-offs)”, “national champion”, and “national team member”), (iv) participants’ self-perceived competence on nutrition and dietary supplementation (subjective opinion on knowledge - S/KN&DS), (v) participants’ knowledge on nutrition and dietary supplementation (objective evaluation of knowledge - O/KN&DS), (vi) the main source of information/knowledge of nutrition and DS (responses included: “I don’t have knowledge on it”, “Coach/physician”, “Formal education [school, club, federation]”, and “self-education [internet, books, magazines, etc.]” ), and (vii) DSU. The O/KN&DS was tested by a questionnaire consisting of 10 questions: (1) The negative side effects of heavy sweating are best remedied by drinking pure water; (2) After a competition day is over, it is better to not eat for 4 h after the competition; (3) Dark yellow urine is an indicator of proper hydration of the body; (4) Recovery drinks consumed after aerobic endurance training should not contain carbohydrates; (5) Large chains of amino acids form carbohydrates; (6) Protein supplementation requires an increased intake of water; (7) Fresh fruits and vegetables are the best sources of high-quality proteins; (8) Beta-alanine is an amino-acid; (9) Carbohydrate drinks should be avoided before matches/games because they encourage urination and, therefore, dehydration; and (10) A decrease in body weight as a result of a single training session indicates dehydration.

Each question was answered in true/false form, and if answered correctly, the participant was given one point (otherwise zero); consequently, the total score ranged from “0” to “10”. The S/KN&DS was evaluated by one question in which participants were asked about their self-perceived knowledge regarding nutrition and DS (responses included: “I have a poor knowledge about it”, “below average”, “average”, “good/very good”). The participants were asked about their DSU with two questions. First, they were asked about their DSU (possible answers were: “Yes, I regularly use DSs”, “Yes, but irregularly/from time to time”, “No, I don’t use DSs”). Those who replied positively to the first question were then asked about their usage of specific types of DSs (vitamins/minerals, carbohydrates, proteins/amino acids, isotonics, iron supplementation, recovery supplements, energy bars, creatine, and other DSs), including the frequency of usage (“regularly”, “from time to time”, “rarely”, “never”). To avoid misinterpretation of certain DS types, several most common examples for each specific type of DS were specified in each question. For this purpose, we used and named the most popular DS brands in Southeastern Europe. Additionally, one of the investigators was at the athletes’ disposal during the testing to answer any possible questions. This questionnaire was previously applied and validated in similar samples, including team sport players [12, 20].

Participants were tested in groups of five or more. Each participant was secured in their own personal space to ensure that they could not communicate with the other participants and that only they could see their answers. Prior to testing, all participants were informed that the testing was anonymous, that they could refuse to participate, that they could leave some questions and/or the entire questionnaire unanswered, and that the returning of the questionnaire would be considered as their consent to participate in the study; this information was also clearly specified in the questionnaire. The testing lasted less than approximately 10 min, and after completing the survey, the participants placed their questionnaires in a closed box that was opened the day after the testing. The study fulfilled all necessary ethical standards of the Declaration of Helsinki for Research on Human Subjects 1989 and was approved by the Faculty of Kinesiology (University of Split, Croatia) ethical board (EBO 10/09/2014–1).

Different sport-specific forms of the questionnaires (i.e., only sport-specific questions were modified for the different sports of interest) were previously studied for reliability and validity in athletes involved in different sports, and the results are presented in detail elsewhere [11, 12, 20]. For the purpose of this study, a convenient sample of 33 players (12 females) was tested twice in a time span of 15 days to identify the test-retest reliability of questionnaires. The correlation coefficient for age was almost perfect (Pearson’s r = 0.99), the correlation was very high for the O/KN&DS (Pearson’s r = 0.86), and the correlation was also very high for the S/KN&DS (Spearman’s r = 0.91). The percentage of identical responses for the question on gender was 100, and 95% of the responses were identical for the question on DSU, all indicating the high test-retest reliability of the measuring tools used in this study.

## Statistical analyses

Statistics included means and standard deviations for age, O/KN&DS, frequency (F) and percentages (%) for the remaining variables. Differences between gender and sports were identified by a Chi-square test, a Kruskal-Wallis analysis of variance, or a one-way analysis of variance (ANOVA), depending on the parametric/nonparametric nature of the variables. The associations between O/KN&DS and S/KN&DS were evidenced by Spearman’s correlation. To identify relations between the studied variables (predictors) and criterion (DSU), a multinomial regression analysis was applied. The criterion included three responses (Regular-DSU; Irregular-DS; No-DSU), and No-DSU was used as the reference value. Although we were mostly interested in the associations between S/KN&DS and O/KN&DS and the criterion (e.g., DSU), all predictors were simultaneously included in the regression calculation to control for the possible confounding effects of different variables. Regression analyses were calculated for the total sample (all players) and separately for basketball, soccer, volleyball, and handball players. For the purpose of the regression analysis, all variables but “gender” were observed as continuous. The odds ratio (OR) with corresponding 95% confidence intervals (95% CI) were reported. Statistica ver. 13.0 (Dell Inc., Tulsa, OK) was used for all calculations, and a *p*-value of 0.05 indicated significance.

## Results

In the total sample, 12.7% of the players consumed DSs regularly, and an additional 35.6% reported the occasional use of DSs, with no significant difference between genders (Chi-square = 1.46, *p* = 0.48). Significant differences in DSU were noted among the sports (Chi-square = 26.67, *p* < 0.01), with the highest prevalence of DSU in basketball players (53% users), followed by handball players (49% users), soccer players and volleyball players (46% users) (Table 1).

**Table 1:** Dietary supplement use by gender or team-sport, with differences between corresponding groups (Chi Square)

	Dietary supplement use						
	Regular		From time to time + Rarely		No		Chi square
	F	%	F	%	F	%	(*p*)
Males	66	11.9%	208	37.4%	282	50.7%	1.46 ^¥^ (0.48)
Females	48	13.5%	120	33.7%	188	52.8%	
Basketball	46	20.2%	74	32.5%	108	47.4%	26.67 ^#^ (0.01)
Soccer	30	9.3%	120	37.0%	174	53.7%	
Volleyball	6	3.9%	66	42.9%	82	53.2%	
Handball	32	15.5%	68	33.0%	106	51.5%	

^¥^indicates Chi square differences (*n* = 912, df = 2) calculated between genders for dietary supplement use

^#^indicates Chi square differences (*n* = 912, df = 6) calculated among sports for dietary supplement use

**Fig. 1:**
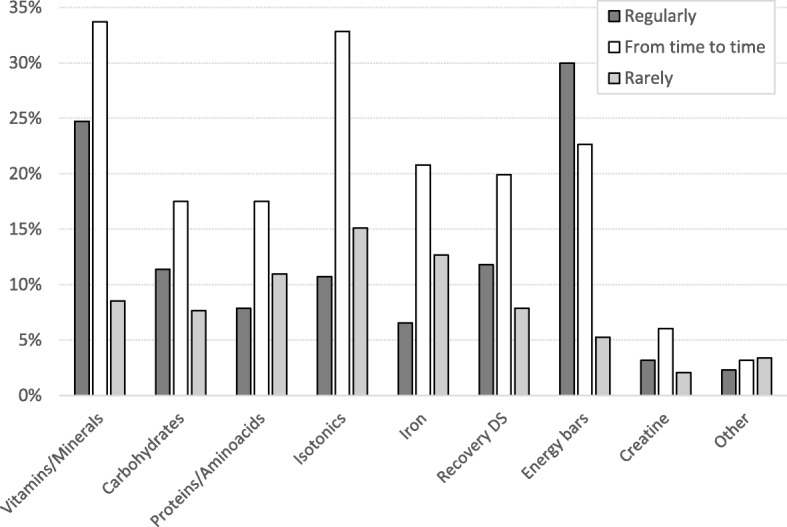
The usage of specific dietary supplements (DS) in team-sport players

The consumption of specific DSs in team-sport players is presented in Fig. 1. Briefly, vitamins/minerals were most commonly used (67% players used them at least “rarely”), followed by isotonics (59%), energy bars (58%), iron (40%), recovery supplements (40%), carbohydrates (37%), proteins/amino acids (36%), creatine (11%), and other supplements (i.e., ginseng, Tribestan, omega-3, echinacea; 9%).

The usage of specific DS across sports is presented in Additional file 1: Table S1. With regard to differences among sports, we emphasize that creatine is mostly used in basketball (15% regular/occasional/rare users), followed by handball (14%) and soccer (13%), while it is less prevalent in volleyball (3% of users). Proteins/amino acids were mostly used in basketball (44%) and handball (44%), followed by soccer (33% of users) and volleyball (21% of users). The smallest differences in consumption among sports is evident for energy bars (57, 60, 60 and 51% of users in basketball, soccer, volleyball and handball, respectively).

**Fig. 2:**
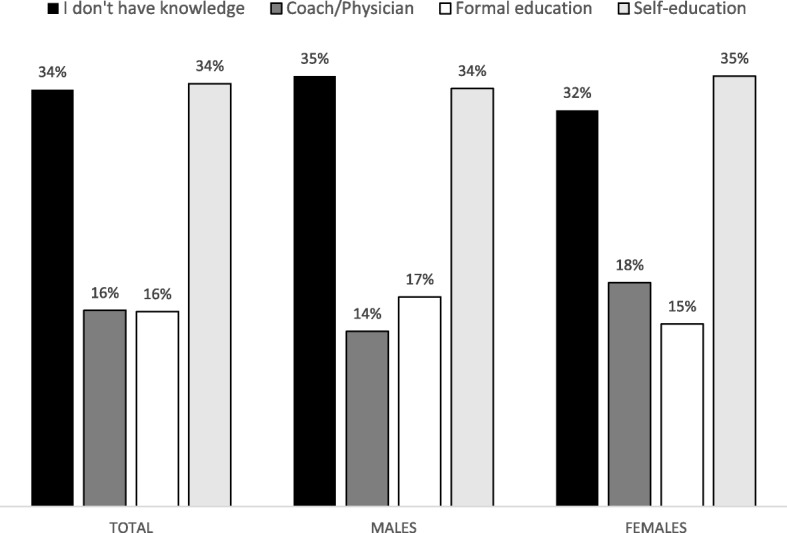
Sources of knowledge about nutrition and dietary supplementation in team-sport players

The main sources of information on nutrition and DS are presented in Fig. 2. In short, the majority of tested athletes declared “self-education” as the most important source of knowledge on nutrition and DS (34%), with no significant differences between genders (Chi square: 2.85, *p* = 0.41).

With an average result of 4.58 ± 2.27, the ANOVA did not reveal significant differences among the players of different sports in terms of O/KN&DS (F-test: 1.88, *p* = 0.13). The players of the different team sports differed significantly in S/KN&DS (KW: 48.03, *p* < 0.01), with the highest self-perceived knowledge observed in the volleyball players and the lowest self-perceived knowledge in the soccer players (Table 2).

**Table 2:** Knowledge on nutrition and dietary supplements (O/KN&DS) and self-percieved competency on nutrition and dietary supplements (S/KN&DS) with differences among sports for O/KN&DS (Analysis of Variance – ANOVA), and S/KN&DS (Kruskal-Wallis test – KW)

	O/KN&DS^a^		S/KN&DS				
	Mean ± SD	ANOVA (*p*)	Poor	Under-average	Average	Good/very good	KW (*p*)
Basketball	4.69 ± 2.29		106 (46%)	16 (7%)	82 (36%)	24 (11%)	
Soccer	4.27 ± 2.19		196 (60%)	26 (8%)	94 (29%)	10 (3%)	
Volleyball	4.77 ± 2.13	1.88	48 (31%)	12 (8%)	68 (44%)	26 (17%)	48.03
Handball	4.81 ± 2.27	(0.13)	102 (50%)	28 (14%)	60 (29%)	16 (8%)	(0.01)

^a^Theoretical range for O/KN&DS was from 0 to 10, with 10 indicating the highest score (best knowledge)

The correlation between O/KN&DS and S/KN&DS was low, although it was statistically significant when calculated for the total sample of participants and females (*r* = 0.10 [*p* < 0.01], *r* = 0.28 [*p* < 0.01], *r* = 0.01 [*p* = 0.81] for the total sample, females, and males, respectively).

When a multinomial regression analysis was performed for all players (e.g., not dividing them according to sport), age was negatively related to regular DSU (OR: 0.91 [95% CI: 0.85–0.98]), indicating a higher prevalence of regular DSU in younger players. Higher odds for regular DSU and irregular DSU were found for those who were convinced of their advanced knowledge on nutrition and DSs (i.e., those with high scores for S/KN&DS (OR: 1.67 [95% CI: 1.44–1.92], and OR: 1.11 [95% CI: 1.01–1.22], for regular- and irregular-DSU, respectively). Additionally, DSU was more prevalent in those with better scores on O/KN&DS (OR: 1.15 [95% CI: 1.04–1.26], and OR: 1.08 [95% CI: 1.01–1.16] for regular- and irregular-DSU, respectively).

Regular DSU was more prevalent in basketball players who had higher scores for S/KN&DS (OR: 2.51 [95% CI: 1.85–3.42]) and those who achieved higher scores for O/KN&DS (OR: 1.25 [95% CI: 1.04–1.51]). Additionally, in basketball players, the S/KN&DS was positively correlated with irregular consumption of DSs (OR: 1.48 [95% CI: 1.20–1.83]). In soccer players, irregular DSU was less prevalent among older players (OR: 0.87 [95% CI: 0.75–0.98]) and players who achieved better competitive results (OR: 0.13 [95% CI: 0.05–0.38]). The achieved competitive result was the only significant factor that correlated with DSU in volleyball players, and volleyball players who achieved better results were less prone to regular DSU (OR: 0.33 [95% CI: 0.13–0.84]). For handball players, regular DSU was more prevalent in younger players (OR: 0.83 [95% CI: 0.72–0.95]) and those who reported higher S/KN&DS (OR: 2.12 [95% CI: 1.55–2.90]). Additionally, male handball players were more prone to irregular DSU than their female peers (OR: 2.21 [95% CI: 1.08–4.52]), (Table 3).

**Table 3:** Results of multinomial regression calculations for dietary supplement use (DSU) as criterion variable, with non-usage of the dietary supplements as reference value

	Regular-DSU	Irregular-DSU
	OR (95%CI)	OR (95%CI)
TOTAL SAMPLE (*N* = 914)		
Age	0.91 (0.85–0.98)	0.97 (0.93–1.02)
Competitive result	1.00 (0.99–1.00)	1.00 (1.00–1.00)
S/KN&DS	1.67 (1.44–1.92)	1.11 (1.01–1.22)
O/KN&DS	1.15 (1.04–1.26)	1.08 (1.01–1.16)
Gender		
Male	0.94 (0.61–1.45)	1.15 (0.85–1.54)
Female	REF	REF
BASKETBALL (*N* = 228)		
Age	0.96 (0.85–1.1)	1.01 (0.93–1.1)
Competitive result	1.18 (0.54–2.60)	1.29 (0.73–2.28)
S/KN&DS	2.51 (1.85–3.42)	1.48 (1.20–1.83)
O/KN&DS	1.25 (1.04–1.51)	1.12 (0.97–1.29)
Gender		
Male	1.49 (0.65–3.41)	0.92 (0.48–1.74)
Female	REF	REF
SOCCER (*N* = 324)		
Age	1.22 (0.89–1.69)	0.86 (0.75–0.98)
Competitive result	1.67 (0.19–14.70)	0.13 (0.05–0.38)
S/KN&DS	1.73 (0.76–3.96)	0.85 (0.67–1.06)
O/KN&DS	0.83 (0.53–1.30)	1.11 (0.93–1.32)
Gender		
Male	0.76 (0.11–5.15)	1.15 (0.56–2.37)
Female	REF	REF
VOLLEYBALL (*N* = 154)		
Age	1.06 (0.91–1.22)	1.03 (0.94–1.13)
Competitive result	0.33 (0.13–0.84)	0.73 (0.47–1.13)
S/KN&DS	1.12 (0.85–1.47)	1.13 (0.95–1.34)
O/KN&DS	1.06 (0.88–1.28)	1.06 (0.95–1.19)
Gender		
Male	0.73 (0.32–1.65)	0.82 (0.50–1.34)
Female	REF	REF
HANDBALL (*N* = 206)		
Age	0.83 (0.72–0.95)	0.95 (0.87–1.03)
Competitive result	1.08 (0.64–1.82)	1.08 (0.64–1.82)
S/KN&DS	2.12 (1.55–2.90)	0.97 (0.77–1.22)
O/KN&DS	1.09 (0.91–1.30)	1.11 (0.97–1.26)
Gender		
Male	0.62 (0.25–1.56)	2.21 (1.08–4.52)
Female	REF	REF

LEGEND: Age – age of the players, Competitive result – the highest competitive result the athlete achieved in sport, S/KN&DS – self-perceived competence on nutrition and dietary supplementation, O/KN&DS – evaluation of knowledge on nutrition and dietary supplementation, REF – reference value in regression calculation

## Discussion

There were several important findings in this study. First, the DSU was lower in older and more successful players. In addition, both self-perceived and objectively evaluated KN&DS were related to DSU, and therefore, the initial study hypothesis was accepted. However, the S/KN&DS was a stronger predictor of DSU than the O/KN&DS, while the correlations between KN&DS and DSU were more evident in athletes who played team sports with a higher prevalence of DSU.

Previous studies correlated DSU with athletes’ age, but the results were not consistent. For example, our finding of a higher prevalence of DSU in younger and less successful athletes is in agreement with the results of previous related studies performed on sailing athletes and rugby players from the territory of Southeastern Europe [12, 26]. On the other hand, it is in certain disagreement with the findings summarized in the meta-analysis of Knapik et al. where the authors concluded that DS is more prevalent in older athletes [14]. However, the differences may be at least partially explained by the fact that practically all studies, including this one, where higher prevalence of DS is reported for “younger” athletes actually observed adults (+ 18 years) [12, 26], and therefore, we are not speaking about youth-athletes but rather “younger adults”. On the other hand, studies summarized in a previously cited review in which a higher prevalence of DS was evidenced in older athletes mostly compared “youth” with “adult” athletes [14].

Several factors influenced the increase of DSU in modern sports. Most likely, the DSU has become more prevalent because of (i) an increase in the psycho-physiological demands of sports training and competition and because of (ii) supplement market growth and aggressive advertising [14, 27]. Such aggressive marketing is especially oriented toward athletes who seek every legal edge to improve their performance [14, 28]. As a result, there is a certain possibility that younger players are under the stronger influence of both factors (e.g., increased physical demands and aggressive DS advertising). On the other hand, we may not ignore the fact that younger athletes (i.e., less experienced athletes) are probably less skilled than their more experienced (i.e., older) colleagues. As a result, younger athletes lean more toward DSU simply because of their intention to “bridge the gap” between their current abilities (performance) and desired achievement.

The previous discussion is supported by the established correlation between sport achievement and DSU, where more successful players were identified as being less oriented toward DSU. This outcome is in agreement with previous studies where higher DSU was evidenced in athletes who reported lower competitive success [26]. It is almost certain that the higher prevalence of DSU in less successful players is a direct consequence of their (relative) inferiority in sport achievements. Supportively, studies have already confirmed that athletes who are not satisfied with their achieved competitive results will try to improve their capacities by using different techniques [12]. While one of the central motives of DSU in sport is its direct or indirect influence on sport performance, the negative correlation between DSU and achieved-sport result is actually logical [16, 29]. Therefore, proper knowledge about DSs is essential, highlighting the central problem identified in this study (e.g., identifying the association between KN&DS and DSU) as particularly important.

Although the practice of DSU is actually ancient (i.e., historical evidence notes usage even in ancient Olympians), the physiological and psychological demands of sport participation have increased exponentially over the last few decades, coinciding with increased DSU in athletes [17, 28, 30]. Additionally, modern athletes are often in out-of-home situations, travel frequently, consume nonfamiliar foods, train and compete in different climates, etc. These habits disturb usual and convenient food consumption and alter appropriate nutrient intake, which frequently results in DSU [26]. Therefore, proper knowledge of the possible ergogenic effects of DSs, the importance of DSs in the recovery process, and the potential side effects of DSs are crucial for the proper and safe usage of DSs in athletes [20, 31–33]. Consequently, the positive correlation between O/KN&DS and DSU established here is encouraging.

On the other hand, it is clear that some athletes who consume DSs overrate their knowledge on nutrition and DS, which is evidenced by the low correlation between O/KN&DS and S/KN&DS (r: 0.10). Almost certainly, the lack of objective knowledge puts those athletes who non-objectively perceive their knowledge on DS as high in danger of inappropriate usage of DS and possible detrimental consequences [25, 34]. Therefore, special efforts are needed to increase the level of knowledge on DS in athletes who are not objective about their expertise on the problem. The importance of systematic and organized education is clearly supported by the fact that the majority of athletes declared “self-education” as the main source of information about nutrition and DS (Fig. 2). Although self-education may be a potentially valuable type of life-long learning, it should not be a main source of information on nutrition and DS issues. Namely, only properly educated athletes will be able to objectively evaluate information obtained from different informal sources (i.e., internet, magazines, food stores) and consequently will be less vulnerable to potential misinformation [35].

The previous discussion is even more important because individuals who overestimate their own KN&DS will likely not improve their knowledge on these topics in the future because of the specific cognitive mechanism known as the “anchoring effect” [36]. In short, the “anchoring effect” is a type of cognitive bias that causes individuals to focus on the first available piece of information (the “anchor”) given to them when making decisions. In this case, athletes with high self-perceived knowledge will be “anchored” by their self-rated knowledge on a topic (i.e., S/KN&DS).

Interestingly, sociopsychological studies have clearly noted that the anchoring effect is moderated by the level of “true knowledge on a problem”, and advanced knowledge decreases the anchoring effect [37]. As a result, we may expect that athletes with high O/KN&DS scores will self-decide to improve their knowledge and awareness of nutrition and DS in the future. On the other hand, athletes with low O/KN&DS scores and high S/KN&DS scores will likely not feel an urgency to improve their knowledge of nutrition and DS through self-education. It is more likely that their knowledge should be transcended through systematical and mandatory educational programs organized by responsible institutions (i.e., sport teams, national/regional sporting federations, and public-health authorities).

Our results indicated sport-specific associations between the studied variables, with stronger correlations between predictors and DSU in sports with a higher prevalence of DSU in athletes. This finding emphasizes the necessity of sport-specific investigation of DSU and of similar topics. Namely, when investigating correlations between certain behaviors and practices in sports (i.e., prevalence of DS, doping-related behaviors, counselling practice), some studies have analyzed athletes involved in different sports and sport disciplines as a homogenous sample of participants [14, 38, 39]. Moreover, different sports often vary in the investigated factors, including predictors (i.e., sociodemographic, sport-specific factors, and knowledge) and criteria (i.e., variables of behavior and/or practice) [40, 41]. Therefore, analyzing athletes involved in different sports as a homogenous sample without acknowledging sport specifics will probably lead to confounding effects in the studied factors. Such effects will consequentially limit the applicability of an analysis in real-sport settings.

The previously discussed findings on the specific associations between subjective and objective evaluation of KN&DS are novel to some extent and therefore make possible the discussion of one specific topic that is not directly related to the aim of the study. The studies that have been conducted so far have reported a positive correlation between DSU and potential and/or current doping behavior in athletes [42, 43]. Consequently, athletes who consume DSs are often targeted as being “vulnerable to doping”. Controversially, in other studies, knowledge on nutrition and DSs was found to be protective against doping behavior [20], while here, we found a correlation between KN&DS and DSU. Overall, we may determine a certain possibility of higher doping likelihood, specifically in athletes who use DSs but subjectively judge their KN&DS as high. This determination could reconcile the different findings of reports in which a higher susceptibility to doping was observed in DS users and opposed reports (with lower susceptibility to doping in DS users) [20, 42, 43]. The importance of these issues makes systematic investigations on this topic warranted.

### Limitations and strengths

This study included only athletes involved in team sports from one specific region (e.g., Southeastern Europe) during the competitive season. Additionally, we identified sport-specific associations among the studied factors. Therefore, the results are generalizable to similar samples of athletes in similar circumstances. The cross-sectional design is another important limitation of the study because it does not allow interpretation of cause-effect relationships between variables. Further, in this study power-bars and isotonic drinks are observed as dietary supplements, the list of DSs observed in this study was limited to those most frequently used in the region which may partially skew the results. Therefore, future studies should additionally focus on DS types not observed herein.

This is one of the first studies that systematically studied and objectively compared evaluated and self-perceived knowledge on nutrition and DSs and their potential correlations with DSU in athletes involved in four Olympic team sports. Additionally, important strengths of this investigation included a relatively large sample of participants with a high competitive level from a specific sociocultural environment (i.e., in a region in which the studied sports are the most popular types of sports, a sport-specific design, and the use of previously validated measurement tools.

## Conclusion

In conclusion, our results show relatively stable associations between KN&DS and DSU in team-sport athletes, and athletes who had higher scores for both measures of KN&DS were more likely to consume DSs. However, because the associations were considerably stronger for “subjectively” than for “objectively” evaluated KN&DS, sport authorities should be informed about the necessity of systematic and targeted education for athletes about sport nutrition and DSs. This would be particularly important in sports with a relatively high prevalence of DSU. Additionally, special attention is needed for athletes who self-perceive their knowledge of sport nutrition and dietary supplementation as high. Namely, while the correlation between objective and subjective evaluation of KN&DS was relatively weak (less than 3% of common variance), there is a clear risk for inappropriate usage of DSs, especially with regard to the fact that the majority of studied athletes declared “self-education” as the primary source of information on nutrition and DS.

## Additional file

Additional file 1: **Table S1.** The usage of specific dietary supplements (DS) in each of the studied team-sports in players from southeastern Europe. (DOCX 13 kb)

## Data Availability

The datasets used and/or analyzed during the current study are available here: https://www.dropbox.com/s/p26rgkghrwp0i41/PODACI.sav?dl=0

## Abbreviations

95%CI: 95% confidence interval
ANOVA: One way analysis of variance
DS: Dietary supplementation
DSU: Dietary supplement use
KW: Kruskal-Wallis analysis of variance
O/KN&DS: Objectively evaluated (tested) knowledge on nutrition and dietary supplementation
OR: Odds ratio
S/KN&DS: Subjective self-perceived knowledge on nutrition and dietary supplementation
