# Supplementary material for: What drives athletes toward dietary supplement use: objective knowledge or self-perceived competence? Cross-sectional analysis of professional team-sport players from Southeastern Europe during the competitive season
Source: J Int Soc Sports Nutr. 2019 Jun 14;16:25. doi: 10.1186/s12970-019-0292-9 (PMC6570862; doi:10.1186/s12970-019-0292-9)
Supplement: Supplementary file 1 — Table S1. The usage of specific dietary supplements (DS) in each of the studied team-sports in players from southeastern Europe. (DOCX 13 kb) [file 12970_2019_292_MOESM1_ESM.docx]

Supplementary table 1

The usage of specific dietary supplements (DS) in each of the studied team-sports in players from southeastern Europe

|  | **Basketball** | | |  | **Soccer** | | |  | **Volleyball** | | |  | **Handball** | | |
| --- | --- | --- | --- | --- | --- | --- | --- | --- | --- | --- | --- | --- | --- | --- | --- |
|  | Regularly | From time to time | Rarely |  | Regularly | From time to time | Rarely |  | Regularly | From time to time | Rarely |  | Regularly | From time to time | Rarely |
| Vitamins/minerals | 34.2% | 35.1% | 4.4% |  | 20.9% | 33.8% | 11.7% |  | 20.8% | 27.3% | 11.7% |  | 23.3% | 36.9% | 11.7% |
| Carbohydrates | 12.3% | 21.9% | 8.8% |  | 12.3% | 14.2% | 5.5% |  | 11.7% | 14.3% | 6.5% |  | 8.7% | 20.4% | 10.7% |
| Proteins/Aminoacids | 13.2% | 14.9% | 15.8% |  | 5.5% | 20.3% | 8.0% |  | 2.6% | 11.7% | 6.5% |  | 9.7% | 20.4% | 13.6% |
| Isotonics | 9.6% | 33.3% | 14.9% |  | 11.1% | 36.9% | 16.0% |  | 10.4% | 23.4% | 16.9% |  | 11.7% | 33.0% | 12.6% |
| Iron | 5.3% | 20.2% | 17.5% |  | 11.7% | 24.0% | 11.7% |  | 2.6% | 18.2% | 6.5% |  | 2.9% | 18.4% | 15.5% |
| Recovery DS | 13.2% | 20.2% | 7.0% |  | 12.3% | 22.8% | 8.0% |  | 14.3% | 16.9% | 7.8% |  | 7.8% | 17.5% | 8.7% |
| Energy bars | 33.3% | 7.0% | 16.7% |  | 28.9% | 25.2% | 5.5% |  | 28.6% | 27.3% | 3.9% |  | 29.1% | 21.4% | 3.9% |
| Creatine | 9.0% | 6.0% | 0.0% |  | 7.0% | 4.0% | 0.0% |  | 2.0% | 3.0% | 0.0% |  | 1.0% | 9.0% | 3.0% |
| Other | 3.0% | 5.0% | 1.0% |  | 1.0% | 2.0% | 0.0% |  | 3.0% | 5.0% | 1.0% |  | 0.0% | 1.0% | 2.0% |
